# Assessment of the effect of *Enteromorpha prolifera* on bacterial community structures in aquaculture environment

**DOI:** 10.1371/journal.pone.0179792

**Published:** 2017-07-25

**Authors:** Guorong Lin, Fulin Sun, Chunzhong Wang, Li Zhang, Xinzhong Zhang

**Affiliations:** 1 College of Environmental and Biological Engineering, Fujian Provincial Key Laboratory of Ecology-toxicological Effects & Control for Emerging Contaminants, Putian University, Putian, China; 2 State Key Laboratory of Tropical Oceanography, Daya Bay Marine Biology Research Station, South China Sea Institute of Oceanology, Chinese Academy of Sciences, Guangzhou, China; 3 Putian Institute of Aquaculture Science of Fujian Province, Putian, China; 4 Putian Oceanic and Fishery Enviormental Monitoring Station, Putian, China; 5 School of Life Sciences, Nantong University, Nantong, China; National Taiwan Ocean University, TAIWAN

## Abstract

In recent years, *Enteromorpha prolifera* blooms had serious impacts on costal environments and fisheries in China. Nevertheless, the effects of *E*. *prolifera* on microbial ecology remain unknown. In this study, for the first time, an Illumina sequencing analysis was used to investigate bacterial communities in source water, aquaculture ponds with *E*. *prolifera*, and an aquaculture pond in which *E*. *prolifera* -free. Principal coordinate and phylogenic analyses revealed obvious differences among the bacterial communities in the pond water with and without *E*. *prolifera*. Abundant bacterial taxa in the *E*. *prolifera*-containing pond were generally absent from the pond without *E*. *prolifera*. Interestingly, pond water with *E*. *prolifera* was dominated by *Actinomycetales* (> 50%), as well as by anaerobic bacteria in the underlying sediment (*Desulfobacterales* and *Desulfuromonadales* (> 20%). Pond water in which *E*. *prolifera*-free was dominated by *Rhodobacterales* (58.19%), as well as aerobic and facultative anaerobic bacteria in the sediment. In addition, the ecological functions of other dominant bacteria, such as *Candidatus* Aquiluna, *Microcella* spp., and *Marivita* spp., should be studied in depth. Overall, massive growth of *E*. *prolifera* will have serious effects on bacterial communities, and, thus, it will have an important impact on the environment. The novel findings in this study will be valuable for understanding green tides.

## Introduction

Green tides are colossal accumulations of green macroalgae. These tides are associated with eutrophication, and they have major ecological and economic impacts on coastal environments. In recent years, massive blooms of *Enteromorpha prolifera* have become increasingly frequent in the coastal waters of China. These blooms are characterized by their large scale, severe environmental effects, and long durations, which have led to serious impacts on coastal landscapes, fisheries, and tourism in China. In June 2008, the world’s largest green tide, covering approximately 600 km^2^, occurred along the coast of the Yellow Sea in China [1, 2]. The direct economic loss caused by this tide was 1.32 billion yuan.

The excessive growth of some species of green algae, such as *E*. *prolifera* and *Ulva* spp., has been reported in green tide events in many parts of the world [2–5]. Green macroalgal blooms have substantially changed marine community structures and functions, and they have had negative impacts on commercial fish farms by creating anoxic conditions in enclosed environments. These adverse effects are usually associated with shading, biomass decomposition, and anoxia [6]. The multiple adverse effects of macroalgal blooms may thoroughly alter the function and structure of affected ecosystems [7]. Microalgae have been shown to negatively affect other benthic communities [8], microbenthic communities [9], and macrofauna [10]. Nevertheless, the effect of *E*. *prolifera* on aquatic microbial ecology has not been reported.

Aquaculture systems are relatively complex ecosystems that often harbor diverse bacterial communities. Microbial communities play major roles in aquaculture ponds, as they are closely linked with productivity, nutrient cycling, and the nutrition status and disease resistance of cultured animals [11–15]. Microbial processes, both aerobic and anaerobic, impact other water quality factors and the content of inorganic nutrients [14]. Through the activity of heterotrophic decomposers, nitrogen and phosphorus are recycled to stimulate primary production [11].

The general aim of the present study was to analyze bacterial communities in environments with *E*. *prolifera*, as well as in environments in which *E*. *prolifera*-free, using an Illumina MiSeq sequencing analysis. Our results show that the presence of *E*. *prolifera* alters bacterial communities, and they also reveal the nature of the microbial community in the *E*. *prolifera*-free environment. These results are of great significance for studying the interactions between bacteria and *E*. *prolifera*, and they provide information that will help predict and prevent *E*. *prolifera* blooms in aquaculture environments.

## Material and methods

### Study areas and sampling

**Fig 1:**
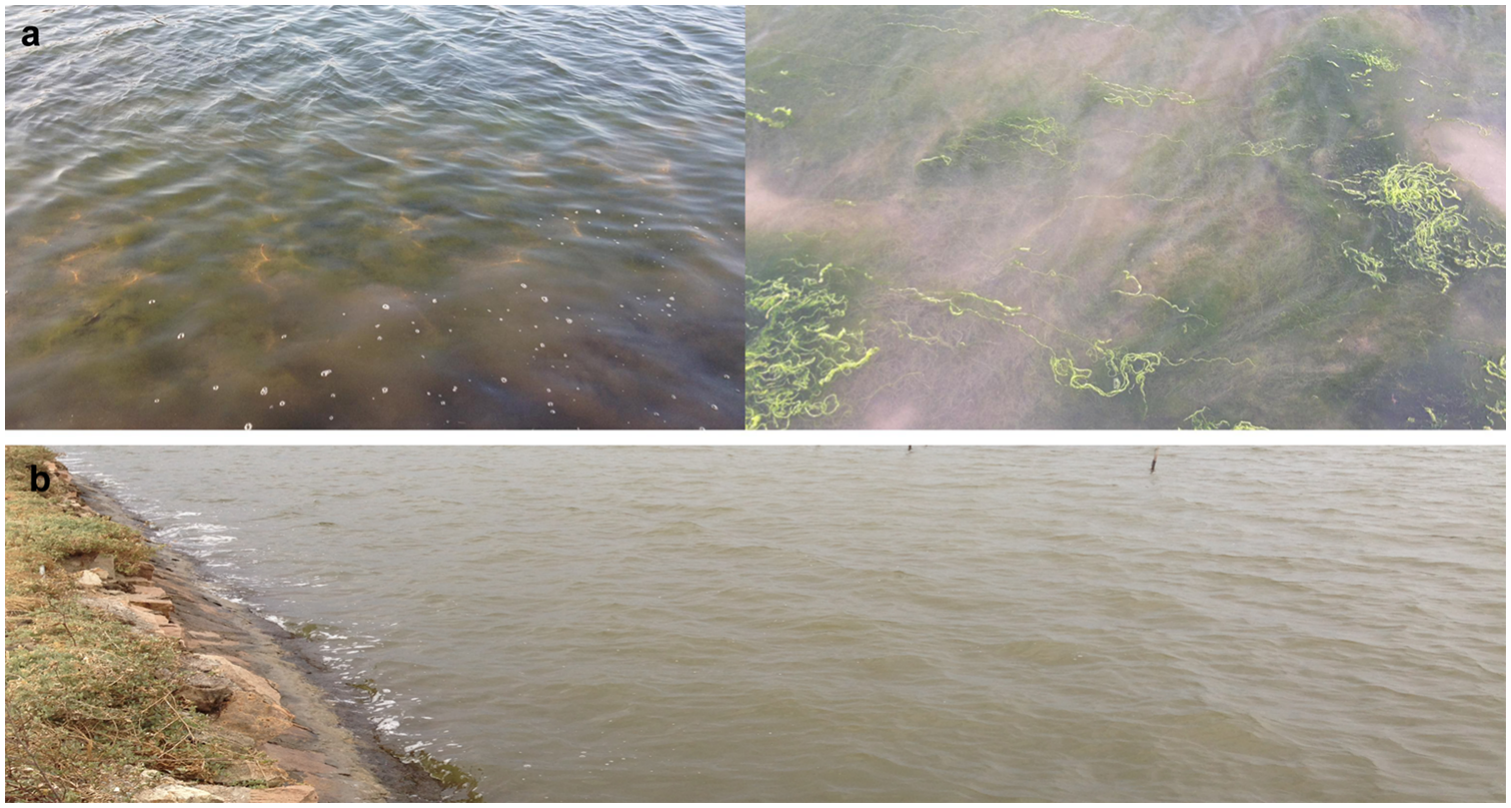
Samples collected from pond water with (a) and without (b) *E*. *prolifera*.

Samples were collected from shrimp farms in Putian, Fujian Province, China. The area of each aquaculture pond is approximately 66,600 m^2^. The investigated culture ponds have the same seawater source, with an inlet. During sampling, outbreaks of *E*. *prolifera* were observed in most of the culture ponds (Fig 1). One pond did not have an occurrence of *E*. *prolifera*. Samples included seawater sources (seawater, SWW; sediment, SWS), three culture ponds with *E*. *prolifera* (water, CPEW1, CPEW2, and CPEW3; sediment, CPES1, CPES2, and CPES3), and one culture pond in which *E*. *prolifera*-free (water, CPW; sediment, CPS). Water samples were collected at 0.5m depth using an organic glass hydrophore. After each sample collection, 500 mL of water was firstly filtered by silk yarn to remove large particles, then was filtered through 0.22-μm filters (EMD Millipore, Billerica, MA, USA) under low (2 m bar) vacuum pressure. After filtration, membranes were put inside 1.5 ml centrifuge tube immediately and frozen in liquid nitrogen. Sediment samples from the ponds were taken at a 0–4-cm depth with a corer sampler. When the sediments were retrieved, air-exposed outer surface of the sediments was discarded and the central cores were taken with a sterile stainless. All of the samples were kept at −80°C before DNA extraction.

### Ethics statement

No specific permits were required for the described field studies. Our study area is not privately owned or protected in any way. Our field studies did not involve endangered or protected species.

### DNA extraction and Illumina MiSeq sequencing

The filters were cut into small pieces and transferred into the extraction tubes using ethanol-flamed forceps and surgical scissors. For each sample, triplicate filters were mixed, and DNA was extracted using the DNA Extraction Kit (Omega, USA) for bacterial communities. One gram (wet weight) of the sediment samples from each frozen section was extracted using a soil DNA extraction kit (Omega, USA) according to the manufacturer’s instructions and sterile techniques to avoid cross contamination.

Then, next-generation sequencing library construction and Illumina MiSeq sequencing were undertaken. Fifty nanograms of DNA was used to polymerase chain reaction (PCR) amplify the V3-V4 hypervariable regions of the bacteria 16S rRNA gene using the primers 319F (5′–ACTCCTACGGGAGGCAGCAG–3′) and 806R (5′–GGACTACHVGGGTWTCTAAT–3′). Indexed adapters were added to the ends of the 16S rRNA amplicons by a limited-cycle PCR. DNA libraries were validated using an Agilent 2100 Bioanalyzer (Agilent Technologies, Santa Clara, CA, USA), and quantified by Qubit and real-time PCR (Applied Biosystems, Carlsbad, CA, USA). DNA libraries were multiplexed and loaded onto an Illumina MiSeq analyzer (Illumina, San Diego, CA, USA) according to the manufacturer’s instructions. Sequencing was performed using a 2 × 250 paired-end configuration, and image analysis and base calling were undertaken by the MiSeq control software of the MiSeq analyzer. All of the sequences can be downloaded from the National Center for Biotechnology Sequence Read Archive Database under the accession number SRR5665810.

### Data analysis

Primer sequences were trimmed from the reads, and reads that contained Ns, were shorter than 400 bp, or had primer mismatches were excluded. Singleton amplicons were removed. Unique sequences were aligned and clustered into operational taxonomic units (OTUs) at a 97% cutoff using the QIIME platform with the UCLUST method. Then, the sequences were assigned to a taxonomy by the RDP classifier with a confidence cutoff of 0.8. Shannon diversity and observed species metrics were assessed using the QIIME platform. A principal coordinate analysis (PCoA) was conducted by computing the unweighted UniFrac distances among the samples using QIIME. Following the output taxonomy file classification, a heatmap was generated to depict the relative percentage of each classification of bacteria (*y*-axis) within each sample (*x*-axis clustering) using the GENE-E module. A taxonomy assignment of tags and OTUs was performed using the Global Alignment for Sequence Taxonomy method.

## Results

### Diversity analysis

A total of 1,321,884 raw reads that correspond to the exact sequences of the samples were obtained. After tag merges and quality control, we obtained a total of 1,280,418 tags (S1 Table). The results revealed that the compositions of the microbial communities were significant different among the different samples (analysis of variance (ANOVA), *p* < 0.01). Diversity entails both taxon richness and evenness, and our results demonstrated that both parameters were highest in the SWW and SWS, and lowest in the CPW and CPS (S1 Table). OTUs and Chao1 estimator supported the aforementioned diversity results, and there were significant differences (ANOVA, *p* < 0.01) between water with and without *E*. *prolifer*a. Our results suggest that the ponds with *E*. *prolifera* had higher bacterial richness and diversity than ponds without *E*. *prolifera*.

### PCoA

**Fig 2:**
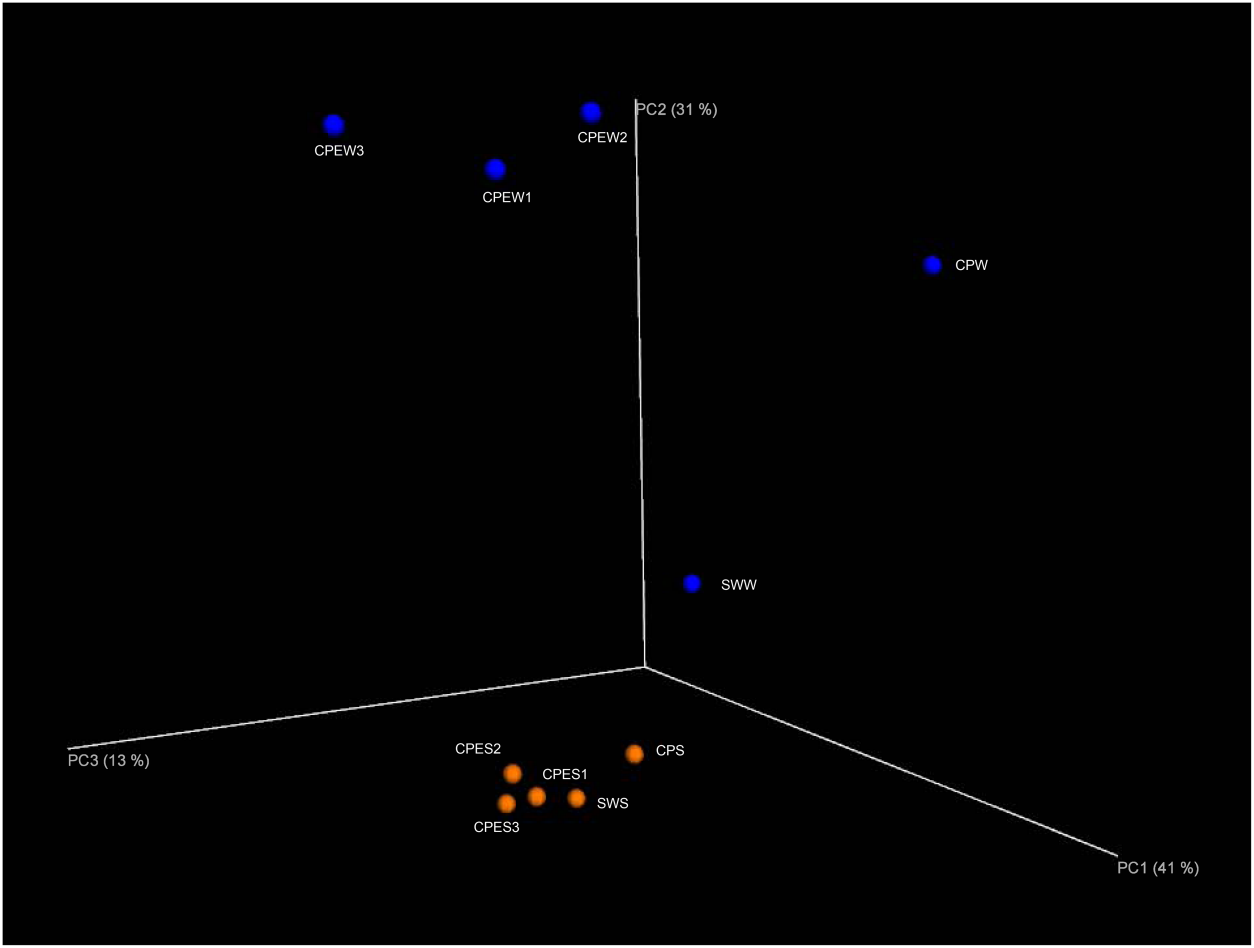
Principal coordinate analysis (PCoA) of unweighted UniFrac distances between pond samples. Blue symbols represent pond water samples. Orange symbols represent pond sediment samples. The given percentages represent the amount of variance explained by the corresponding axis.

To further analyze the effect of *E*. *prolifera* on bacterial community composition (beta diversity), we summarized the unweighted UniFrac distances among the samples via a PCoA (Fig 2). This analysis clustered the microbial communities according to different samples (analysis of similarity (ANOSIM), *p* < 0.001). The clustering results also indicated that groups of pond sediment communities were more similar to each other than to those of the pond water samples. In addition, pond water samples with *E*. *prolifera* clustered together, and they were separated from inlet water and pond water without *E*. *prolifera*, indicating that bacterial communities in the CPEW were affected by *E*. *prolifera*.

### Taxonomic composition

All sequences were classified from phylum to genus according to the program QIIME using the default setting, and 53 different phyla or groups were identified in the samples. Phylogeny more intuitively illustrates community differences among the different samples. Node size is expressed as abundance; the green coverage area represents low abundance; and the red coverage area represents high abundance. Phylogeny displays the development and evolution of bacterial species from the outside seawater to the pond water.

#### Phylum-order taxonomic distribution

**Fig 3:**
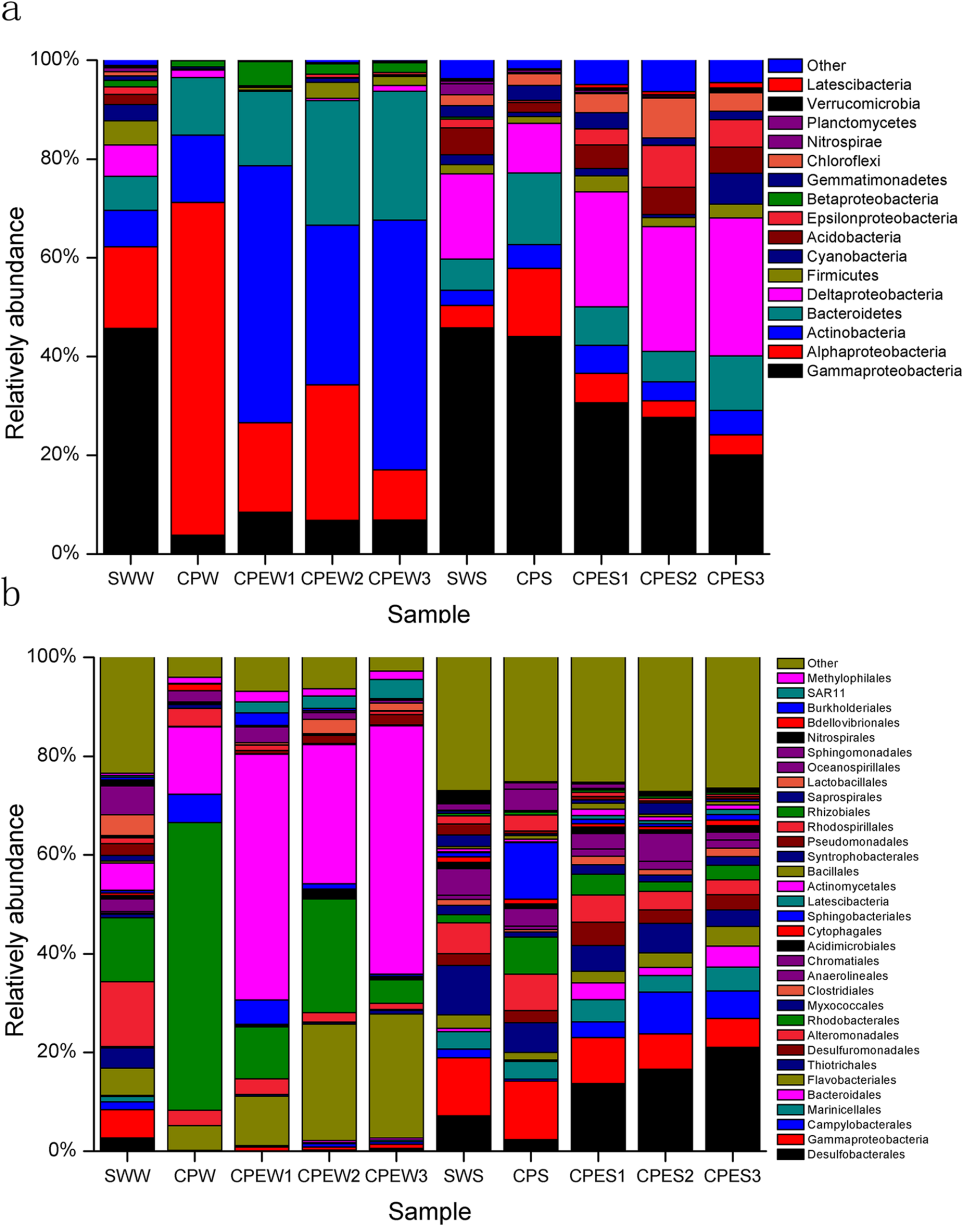
Differences in bacterial communities between the source water (SWW and SWS), pond water with *E*. *prolifera* (CPEW and CPES), and pond water in which *E*. *prolifera*-free (CPW and CPS). a, Phylum taxonomic level; b, Order taxonomic level.

Water samples and sediment samples displayed dissimilar 16S rRNA profiles, even for phylum-order level distributions (Fig 3). The SWW was primarily composed of *Gammaproteobacteria* (45.71%), *Alphaproteobacteria* (16.55%), *Bacteroidetes* (6.89%), and *Actinobacteria* (7.34%), and it was mainly dominated by the orders *Alteromonadales* (13.10%), *Rhodobacterales* (12.97%), *Oceanospirillales* (5.80%), *Actinomycetales* (5.55%), and *Flavobacteriales* (5.51%). The CPEW1, CPEW2, and CPEW3 were primarily composed of *Actinobacteria* (32.35%–52.05%), *Alphaproteobacteria* (10.16–27.14%), *Bacteroidetes* (15.08%–26.10%), and *Gammaproteobacteria* (6.86%–8.50%), and they were mainly dominated by the orders *Actinomycetales* (28.17%–50.29%), *Rhodobacterales* (4.79%–23.02%), and *Flavobacteriales* (9.98%–25.11%). The CPW was mainly composed of *Alphaproteobacteria* (67.42%), *Bacteroidetes* (11.63%), and *Actinobacteria* (13.63%), and it was mainly dominated by the orders *Rhodobacterales* (58.19%), *Actinomycetales* (13.61%), *Sphingobacteriales* (5.78%), and *Flavobacteriales* (5.01%).

Sediment samples had significantly different abundances of bacterial taxa (ANOVA, *p* < 0.01). The SWS had higher levels of *Thiotrichales* (10.03%) and *Chromatiales* (5.42%) than the CPES and CPS. The CPES had higher *Desulfobacterales* (13.73%–21.07%) abundances than the other sediment samples. The CPS had the highest abundances of *Sphingobacteriales* (11.50%) and *Rhodobacterales* (7.47%), while it had a relatively low abundance (2.37%) of *Desulfobacterales*.

#### Genus-level distribution

**Fig 4:**
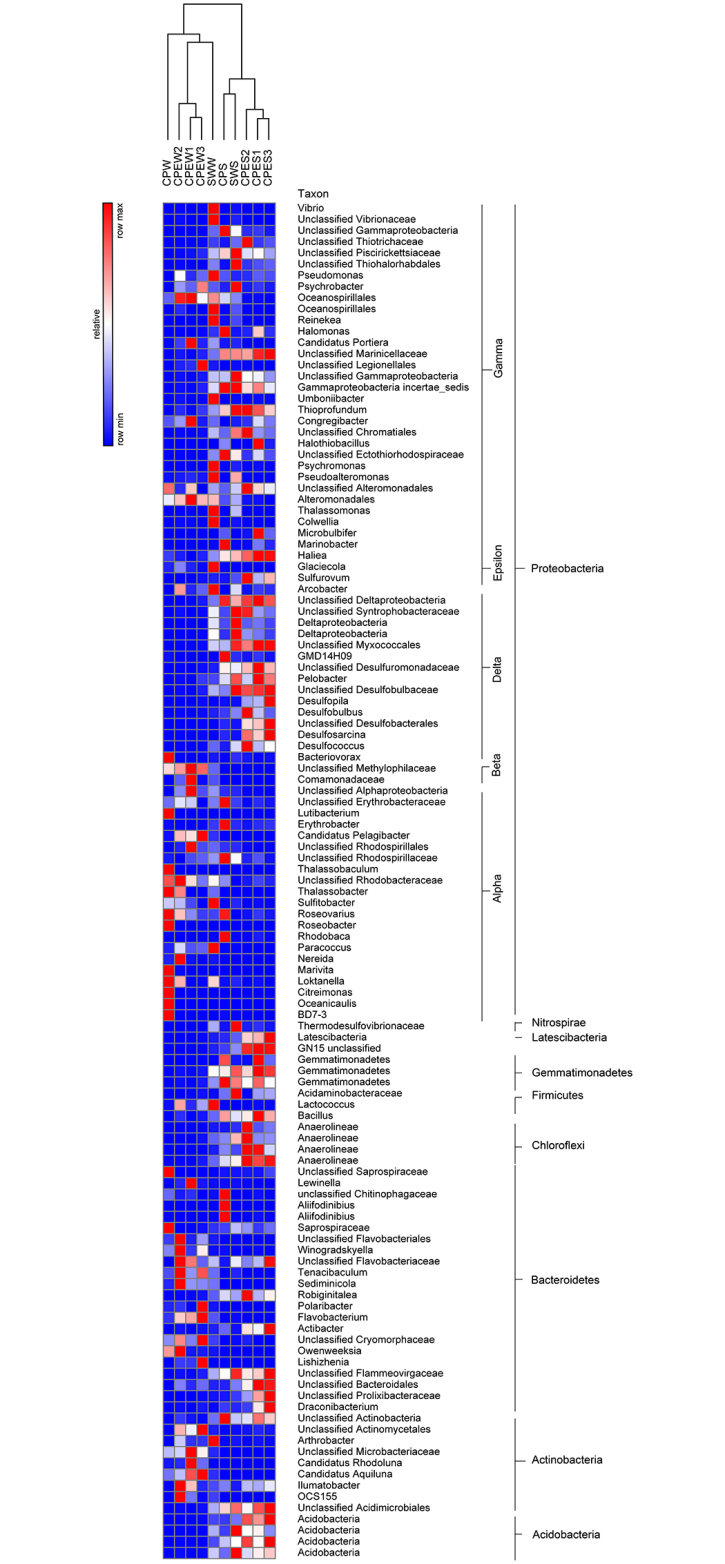
Heatmap showing the phylogenetic distribution between the source water (SWW and SWS), pond water with *E*. *prolifera* (CPEW and CPES), and pond water in which *E*. *prolifera* -free (CPW and CPS). The relative percentage of each bacterial genus (*y*-axis) in each sample (*x*-axis clustering) is shown. Colored bars represent relative percentages.

Heatmaps were used to intuitively display the differences in the relative abundances of different genera (Fig 4). The genera with the highest contribution to the ordination were selected. More than 60% of the total OTUs corresponded to *Proteobacteria* in each library. The most dominant groups of the total *Proteobacteria* tags were *Alphaproteobacteria*, *Deltaproteobacteria*, and *Gammaproteobacteria*. The next most dominant groups of the total bacterial tags were *Bacteroidetes* and *Actinobacteria*. The dominant genera and families in the SWW were *Vibrio* (8.24%), *Rhodobacteraceae* (7.97%), *Lactococcus* (3.90%), and *Piscirickettsiaceae* (3.54%). The CPEW was characterized by *Microbacteriaceae* (11.87%–22.12%), *Candidatus* Aquiluna (8.64–23.02%), and *Rhodobacteraceae* (4.01%–14.41%). The CPW was dominated by *Rhodobacteraceae* (mainly *Marivita* 33.31%), *Rhodobacteraceae* (12.34%), *Roseobacter* (4.95%), *Microbacteriaceae* (8.19%), and *Candidatus* Aquiluna (5.29%). The CPES was dominated by many genera that were affiliated with *Desulfobacterales* (16.15%–22.10%) and *Gammaproteobacteria* incertae sedis (4.81%–7.83%). The CPS was dominated by *Gammaproteobacteria* incertae sedis (10.37%), *Aliifodinibius* (6.00%), *Marinobacter* (5.64%), *Rhodobacteraceae* (4.59%), and *Chitinophagaceae* (3.83%).

### The most abundant bacterial species, and a detrended correspondence analysis (DCA)

**Fig 5:**
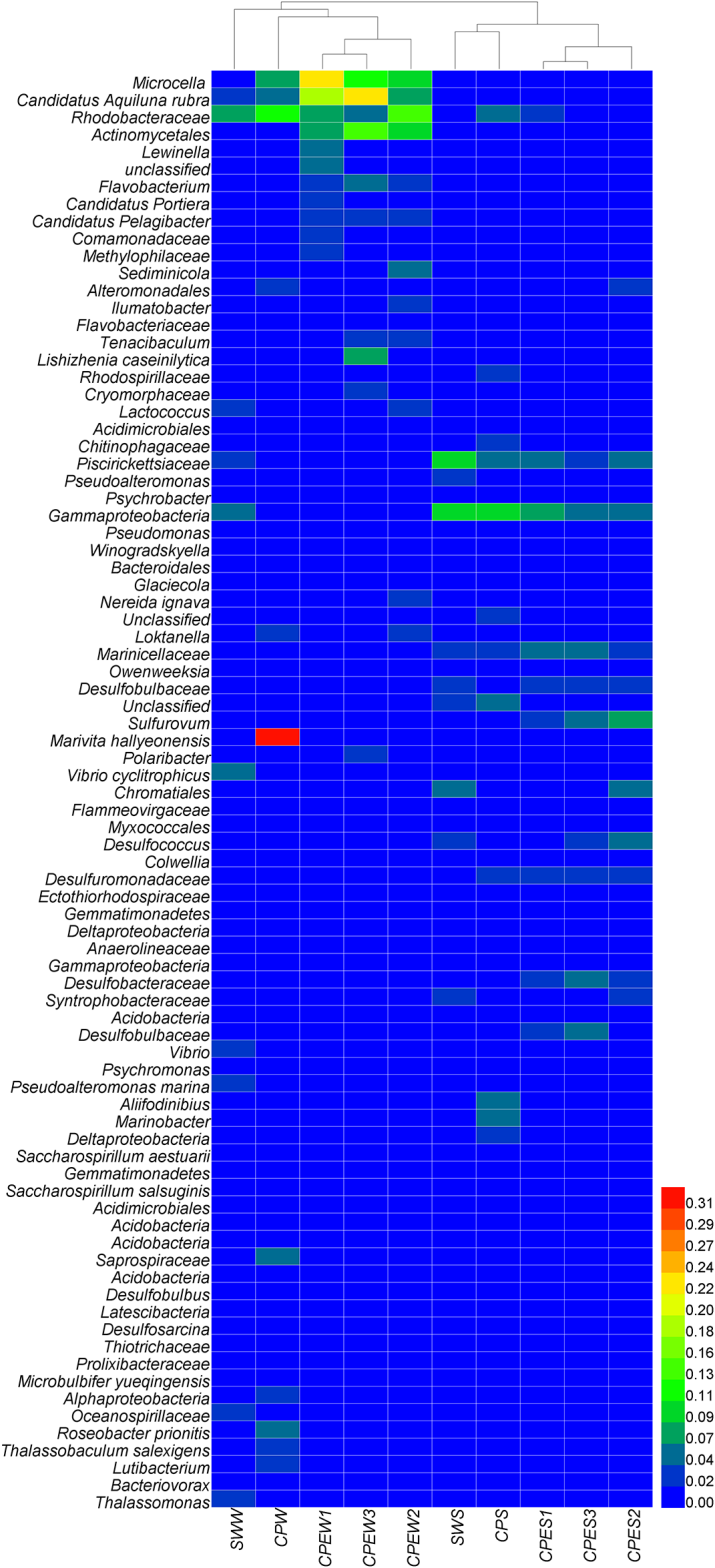
Heatmap showing the differences in the distributions of the most abundant bacterial species (> 1%) among the source water (SWW and SWS), pond water with *E*. *prolifera* (CPEW and CPES), and pond water in which *E*. *prolifera* -free (CPW and CPS). The relative percentage of each bacterial species (*y*-axis) in each sample (*x*-axis clustering) is shown. Colored bars represent relative percentages.

**Fig 6:**
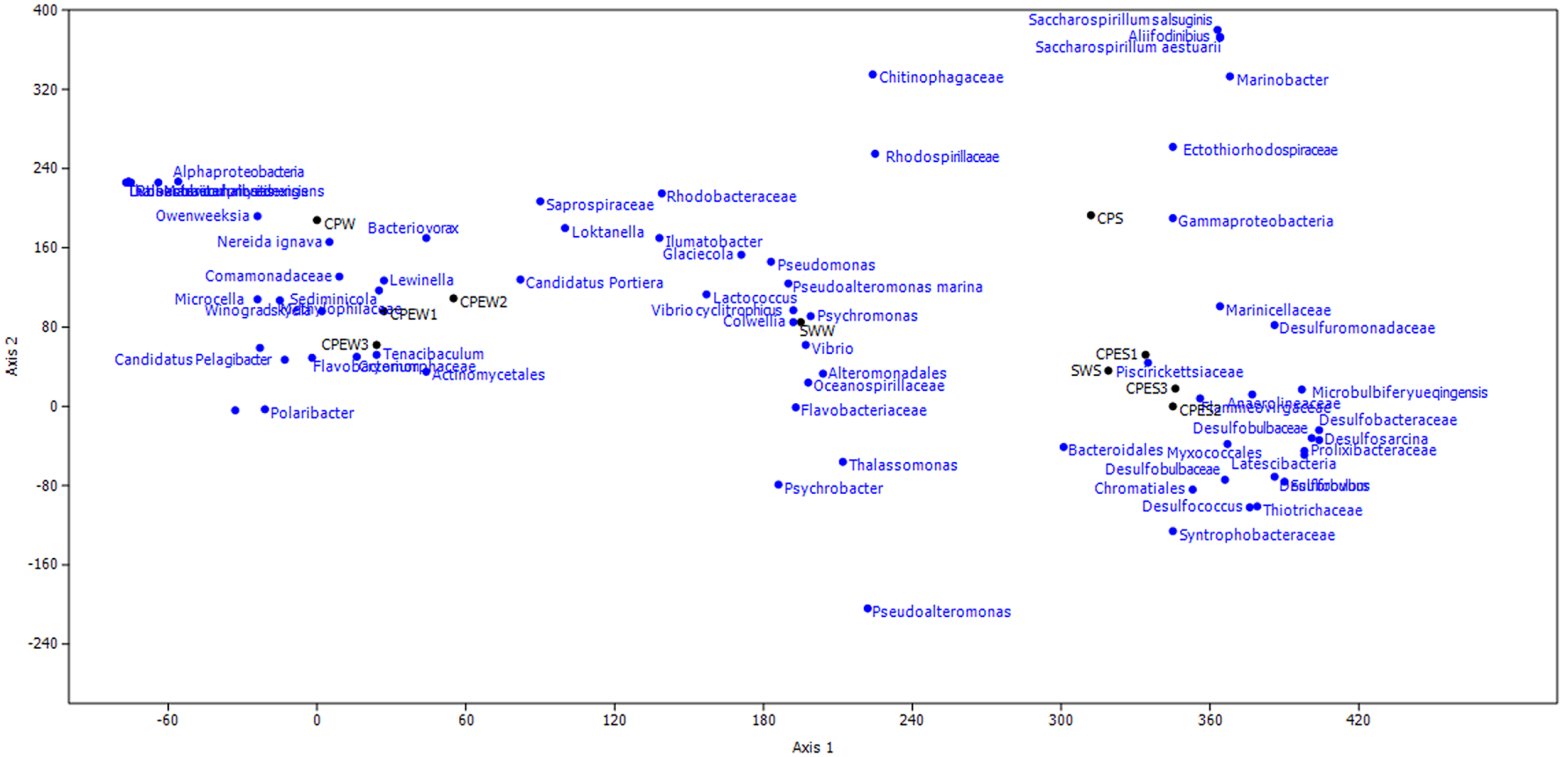
Detrended correspondence analysis (DCA) of bacterial community between the source of water (SWW and SWS), pond water with *E*. *prolifera* (CPEW and CPES), and pond water in which *E*. *prolifera* -free (CPW and CPS).

To explore the effect of *E*. *prolifera* on the bacterial communities, highly abundant (> 1%) species were illustrated with a heatmap (Fig 5) and a DCA (Fig 6). The DCA was performed to discern possible linkages between the samples and the dominant bacterial species. The eigenvalues for the first two multivariate axes were 0.7523 and 0.2094, respectively. The DCA plots showed a clear separation among the samples from different environments. The samples obtained from pond water with *E*. *prolifera* were mainly composed of *Microcella*, *Candidatus* Aquiluna rubra, *Rhodobacteraceae*, and *Actinomycetales*. In the pond water of CPW, the bacterial community was dominated by *Marivita hallyeonensis* (33.31%). Except for common bacterial classes, families, and genera (*Gammaproteobacteria*, *Piscirickettsiaceae*, and *Marinicellaceae*, respectively), some families affiliated with the order *Desulfobacterales*, such as *Desulfuromonadaceae*, *Desulfobulbaceae*, and *Desulfobacteraceae*, dominated the sediment samples from the *E*. *prolifera*-containing environments. These findings indicate that the samples obtained from pond water with *E*. *prolifera* had distinctly different species compositions, compared with the samples from the pond without *E*. *prolifera* (ANOVA, *p* < 0.01).

## Discussion

The purpose of this study was to explore the bacterial diversity from source seawater to the aquaculture environment, and especially to compare bacterial communities in environments with or without *E*. *prolifera*. By applying large-scale 16S rRNA gene sequencing, the microbial populations of these environments were systematically investigated. *Alphaproteobacteria*, *Deltaproteobacteria*, and *Gammaproteobacteria*, *Actinobacteria*, and *Bacteroidetes* were the dominant phyla, and they exhibited distinct distributions among the aquaculture environments (ANOVA, *p* < 0.01). Overall, our results indicate that *E*. *prolifera* changes the species composition and abundance of key bacterial taxa. As a result, *E*. *prolifera* would has a significant impact on the microbial community of aquaculture environments.

The diversity of the microbial community were lower in the culture ponds than those in source water. Similar with our previous study through clone library method [16], aquaculture environment will reduce the bacterial diversity and species number, leading to a simplified structure of the microbial community. That is mainly because culture environment had relatively steady of phy-chemical characteristic than external water body. For pond water with *E*. *prolifera*, bacterial community had higher diversity and richness than those in *E*. *prolifera*-free ponds. Macroalgal biomass not only represents an extremely large source of organic matter, and it is likely to compete with other organisms for inorganic nutrients. In this study, nutrients concentration (nitrogen and phosphorus nutrients) in *E*. *prolifera* ponds were much lower than those in *E*. *prolifera*-free ponds (S1 Fig). Other studies showed that in relatively low-nutrient conditions, bacterial communities exhibit relatively high diversities than those on concentrated media or environment [17, 18]. That should be mainly explained the reason that high bacterial diversity of bacterial community were found in *E*. *prolifera* ponds.

When we consider the dominant individual taxa, a distinct difference was observed between the CPE and CP environments (ANOVA, *p* < 0.01). The most prominent example of this difference is the presence in the CPW and CPS of many species that are rare in the CPEW and CPES samples (Fig 6). This result is in agreement with many studies that showed that phytoplankton affect the microbial community structure [19–21]. Another study suggested that macroalgae species may control their epibiotic bacterial communities [22]. Therefore, the distinct differences of the bacterial communities between the CPEW and CPW were caused by the outbreak of *E*. *prolifera*.

Heterotrophic bacteria were the major microbes in the CPEW, and they were dominated by *Actinomycetales* (49.80%), which mainly included the family *Microbacteriaceae* (22.12%) and *Candidatus* Aquiluna (19.66%). Blast results indicated that these *Actinomycetales* sequences shared 99% similarity with *Microcella* and *Candidatus* Aquiluna rubra (Fig 6). *Candidatus* Aquiluna rubra is represented by an aerobic, red-pigmented strain, which was isolated previously from a eutrophic pond [23]. Genome information suggests that *Candidatus* Aquiluna rubra is a photoheterotroph carrying actinorhodopsin, and that it has the smallest genome ever reported for a free-living member of the *Actinobacteria* [24]. The genome sequence of Actinorhodopsin-encoding marine bacterium suggested that this bacteria could be capable of both carbon fixation and rhodopsin-based phototrophy. This highlight the vast potential adaptation for novel metabolic strategies involving rhodopsins [25]. This high potential for photoheterotrophy could lead to an enhanced carbon turnover and/or nutrient acquisition and might be relevant also in terms of carbon sequestration through the microbial carbon pump [26]. *Microcella* is a Gram-positive, chemoorganotrophic bacterium [27]. Thus, the high abundances of *Candidatus* Aquiluna rubra and *Microcella* that were observed in the environment with *E*. *prolifera* make the ecology and evolution of this lineage interesting. However, information regarding these two bacteria only includes their taxonomic classifications, while their ecological functions have not been reported.

Heterotrophic microbes from *E*. *prolifera* habitats readily use the available dissolved organic matter [28]. However, a recent study showed that the active microbial community was strongly correlated with specific dissolved organic matter molecules, specifically compounds that are easily degradable [29]. The results of Gomez-Consarnau et al. (2012) support the view that specific carbon compounds trigger the growth of certain bacterial strains[30]. When *E*. *prolifera* decays, it produces large amounts of organic matter, which is preferred by certain heterotrophic bacteria. This increases the abundance of these bacteria, which ultimately alters the overall bacterial community. This phenomenon was illustrated by a study that showed that the microbenthic community metabolism clearly shifted to heterotrophic, while the photoautotrophic activity was located in the macroalgal canopy [31]. On the other hand, *E*. *prolifera* water will increase the abundance of *Flavobacteriales* genera, such as *Sediminicola*, *Flavobacterium*, *Tenacibaculum*, *Owenweeksia* and *Winogradskyella* (Fig 4). Some species of these genera are potential pathogens [32–34], and increase the infection chance for aquaculture animal. The annual economic losses caused by some *Flavobacterium* pathogens is extremely serious.

The CPW was dominated by *Rhodobacterales* (58.19%), which mainly included *Marivita* (33.31%), *Rhodobacteraceae* (12.34%), and *Roseobacter* (4.95%). The OTUs (31.8% of total sequence) of the genus *Marivita* shared 99% similarity with *M*. *hallyeonensis*. Recently, *M*. *hallyeonensis* has been described as a member of the genus *Marivita*, and it is often found in coastal environments [35]. Although some bacteria, such as *Marivita*, had very high abundances in pond water, their ecological functions remain unknown. There were also many dominant bacterial taxa, such as *Roseobacter*, *Saprospiraceae*, *Thalassobaculum*, *Loktanella*, and *Lutibacterium*, in the pond water of CPW, which were rare in the CPEW pond. In addition, the Rhodobacteraceae family is known to exhibit a diverse range of metabolic activity. These Rhodobacterales genera could undergo aerobic anoxygenic photosynthesis, sulfur oxidation, carbon monoxide oxidation, and DMSP demethylation [36]. They also can improve water quality through reducing concentration of ammonia [37], nitrate [36], and hydrogen sulfide [38]. *Roseobacter* strains can possibly establish an antagonistic beneficial bacterial community in the rearing environment of turbot larva and thereby limit the survival of pathogenic bacteria [39], and can act as probiotic microorganisms in aquaculture environment. In addition, a few roseobacters strains are capable of producing algaecidal substances, so-called roseobacticides [40], which are deadly to some algal strains. Rhodobacteraceae may therefore have played an important role in maintaining the health of the culture system [41], and maybe play important role in inhibiting the occurrence of *E*. *prolifera*. These are very meaningful questions that require in-depth study.

Major obvious effects of macroalgae are due to light attenuation of photosynthetic communities and the production of hypoxic or anoxic conditions. This leads to the production and accumulation of hydrogen sulfide in sediments below macroalgal mats [42, 43]. The CPES had high abundances of sulfate-reducing bacteria (*Desulfobacterales* and *Desulfuromonadales*, > 18% of the total sequences), while few such bacteria were detected in the CPS. *Desulfobacterales* are sulfate-reducing bacteria that reduce sulfates to sulfides to obtain energy. They are strictly anaerobic. Numerous members of the *Desulfuromonadales* are capable of anaerobic respiration by utilizing a variety of compounds as electron acceptors, including sulfur [44]. *Desulfobacterales* (*Desulfobulbaceae* and *Desulfobacteraceae*) have also been identified as the major sulfate-reducing bacteria in sediments beneath intensive aquaculture ponds [45, 46]. Asami et al. (2005) demonstrated that the sulfur cycle is accelerated in sediments beneath shellfish aquaculture ponds, and that certain taxa of sulfur-reducing bacteria were more abundant in these environments [45]. The dominance of anaerobic bacteria might be due to the generation of hypoxic or anoxic conditions by *E*. *prolifera*. This also explains the differences in the relative bacterial abundances, as well as the DCA, between the CPES and CPS samples. These sulfate-reducing bacteria could transform sulfur to hydrogen sulfide under anoxic conditions, which causes serious harm to aquaculture animals and environment.

Regarding bacterial taxa, the CPS was mainly characterized by aerobic and facultatively anaerobic species. These bacterial taxa included *Aliifodinibius*, *Marinobacter*, *Rhodobacteraceae*, and *Chitinophagaceae*, of which a few were detected in the CPES samples. *Aliifodinibius*, a genus of novel Gram-negative rods that are facultatively anaerobic, oxidase-negative, and catalase-positive, is a member of the phylum *Bacteroidetes* [47]. Nevertheless, its ecological function is unknown. *Marinobacter* has nitrous oxide reductase *(nosZ)* genes, and the capability of utilizing nitrite and nitrate to produce N_2_ as the aerobic denitrification product [48]. The CPS was mainly characterized by aerobic bacteria, which can reduce the damage caused by nitrite. These bacteria have the potential to improve the quality of the environment, which is important in aquaculture.

In conclusion, the results from this study demonstrated that there were obvious differences in the bacterial community structures between pond water with and without *E*. *prolifera*. The pond water with *E*. *prolifera* was dominated by *Actinomycetales*, and anaerobic sulfur-reducing bacteria were highly abundant in the underlying sediment. The pond water without *E*. *prolifera* was dominated by *Rhodobacterales*, and aerobic and anaerobic bacteria were highly abundant in the underlying sediment. These findings indicate that the massive growth of *E*. *prolifera* had a significant effect on the microbial community, and that *E*. *prolifera* has the potential to harm the environment.

## Supporting information

S1 Fig Water physico-chemical parameters in pond water with (a) and without (b) *E*. *prolifera*. (TIF)

S1 Table Sample list and sequencing information. (DOCX)
