# Supplementary material for: Assessment of the effect of Enteromorpha prolifera on bacterial community structures in aquaculture environment
Source: PLoS One. 2017 Jul 25;12(7):e0179792. doi: 10.1371/journal.pone.0179792 (PMC5526538; doi:10.1371/journal.pone.0179792)
Supplement: S1 Table — (DOCX) [file pone.0179792.s002.docx]

S1 Table Sample list and sequencing information

| Sample | Raw Data | Valid Data | Valid% | Q20% | Q30% |
| --- | --- | --- | --- | --- | --- |
| SWW | 142395 | 138594 | 97.33 | 95.59 | 86.83 |
| CPW | 125194 | 121876 | 97.35 | 96.24 | 88.11 |
| CPEW1 | 158678 | 154336 | 97.26 | 95.72 | 87.08 |
| CPEW2 | 53025 | 51006 | 96.19 | 95.08 | 85.02 |
| CPEW3 | 77952 | 76301 | 97.88 | 96.35 | 88.46 |
| SWS | 151895 | 146643 | 96.54 | 95.38 | 86.43 |
| CPS | 160861 | 154781 | 96.22 | 95.13 | 85.65 |
| CPES1 | 204877 | 197943 | 96.62 | 95.47 | 86.53 |
| CPES2 | 188232 | 181940 | 96.66 | 95.48 | 86.51 |
| CPES3 | 58775 | 56998 | 96.98 | 95.49 | 86.62 |
